# White matter microstructure and receptive vocabulary in children with cerebral palsy: The role of interhemispheric connectivity

**DOI:** 10.1371/journal.pone.0280055

**Published:** 2023-01-17

**Authors:** Olga Laporta-Hoyos, Kerstin Pannek, Alex M. Pagnozzi, Simona Fiori, Roslyn N. Boyd

**Affiliations:** 1 Queensland Cerebral Palsy and Rehabilitation Research Centre, Faculty of Medicine, The University of Queensland, Brisbane, Queensland, Australia; 2 Australian E-Health Research Centre, CSIRO, Brisbane, Australia; 3 Department of Developmental Neuroscience, IRCCS Stella Maris Foundation, Pisa, Italy; 4 Department of Clinical and Experimental Medicine, University of Pisa, Pisa, Italy; Western University, CANADA

## Abstract

**Background:**

Communication and cognitive impairments are common impediments to participation and social functioning in children with cerebral palsy (CP). Bilateral language networks underlie the function of some high-level language-related cognitive functions.

**Purpose:**

To explore the association between receptive vocabulary and white-matter microstructure in the temporal lobes and the central part of the temporo-temporal bundles in children with CP.

**Materials and methods:**

37 children with spastic motor type CP (mean age 9.6 years, 25 male) underwent a receptive vocabulary test (Peabody Picture Vocabulary Test, PPVT-IV) and 3T MRI. Mean fractional anisotropy (FA) and mean diffusivity (MD) were calculated for the temporal lobes and the interhemispheric bundles traversing the splenium of the corpus callosum and the anterior commissure. Associations between microstructure and receptive vocabulary function were explored using univariable linear regression.

**Results:**

PPVT-IV scores were significantly associated with mean white matter MD in the left temporal lobe, but not the right temporal lobe. There was no association between PPVT-IV and mean white matter FA in the temporal lobes. PPVT-IV scores were not significantly associated with the laterality of these diffusion tensor metrics. Within the corpus callosum, FA, but not MD of the temporo-temporal bundles was significantly associated with the PPVT-IV scores. Within the anterior commissure no equivalent relationship between diffusion metrics and PPVT-IV was found.

**Conclusion:**

Our findings add further understanding to the pathophysiological basis underlying receptive vocabulary skills in children with CP that could extend to other patients with early brain damage. This study highlights the importance of interhemispheric connections for receptive vocabulary.

## 1. Introduction

Cerebral palsy (CP) is the most common cause of physical disability in childhood, affecting 2.11 per 1000 live births worldwide [1]. Multiple comorbidities are commonly associated with CP often adversely affecting the quality of life and health more than the motor impairment itself [2–5]. Comorbidities contribute significantly to the lifetime economic costs of CP [6] and are major impediments to participation [7] and social functioning [8].

More than 55% of children with CP present some level of communication impairment [9, 10]. These impairments are more common in children with bilateral CP and are associated with the motor severity, cognitive functioning, and cortical/subcortical and basal ganglia lesions [9, 10]. Verbal communication, both oral and written, comprises a broad range of language skills including, among others, not only speech production, which is frequently impaired in CP [11] but also receptive vocabulary. Children with CP present with delays in spoken comprehension [12–16] which have been shown to be related to cognitive function [12–16].

Magnetic resonance imaging (MRI) contributes to our understanding of the pathological basis underlying CP and clinical outcomes [17] including our understanding of language reorganization after early brain injury. Conventional structural MRI can provide information on macrostructural abnormalities but has a limited ability to inform on tissue microstructure. Diffusion MRI is a complementary method that measures the diffusion of water molecules to provide insight into tissue microstructure. The tensor model is a common diffusion MRI model from which two metrics are commonly reported as indices of tissue microstructural integrity. The first is mean diffusivity (MD), which provides a measure of the rate of diffusion, which is typically high in regions of lesioned tissue. The second is fractional anisotropy (FA) which indexes the degree to which water diffuses in its most major direction, relative to its perpendicular directions. FA is typically low in lesioned tissue and higher in voxels dominated by a single healthy major white matter tract [18, 19]. A limitation of FA is that it can be difficult to interpret in areas containing multiple crossing fibres [20]. Several studies have assessed the WM microstructural integrity in people with CP by using diffusion imaging. Most of them have explored and identified deficits in the corticospinal tracts and sensory projection fibres while fewer have focused on the commissural and association fibres [21] or have performed more comprehensive whole-brain characterizations of white matter damage in CP [22–25].

In typically developing persons language is predominantly processed in the left hemisphere, while in persons with CP some degree of plasticity has been reported by functional MRI studies [26, 27]. That is, some studies report similar functional activation between right and left hemispheres during language tasks in children with left-circumscribed brain lesions [26, 27]. Additionally, some studies report no laterality effects on specific learning difficulties [28] or within average verbal IQ in children with unilateral CP [29]. In one of these studies, the degree to which children who also presented with right hemisphere accompanying lesions was not reported as the laterality of the lesions was assumed by the side of the hemiplegia [28], while the second reported within average verbal IQ when only analysing children who had left hemisphere observable lesions [29]. Taken together, these reports have been interpreted as evidence of functional equipotentiality. The brain’s ability to reorganize, however, is likely imperfect as left sided brain lesions in children with CP have also been associated with poorer communication skills [30].

In a previous study [31], we found that poorer receptive vocabulary was associated with temporal lobe lesions being distributed toward the left, rather than the right, hemisphere as assessed by the lesion laterality index. After controlling for total lesion load, left-sided unilateral lesions, rather than bilateral lesions, were also found to be associated with poorer receptive vocabulary. These findings could be due to the bilateral lesions directly affecting the left temporal lobe to a lesser degree, and/or due to interhemispheric communication being less disrupted by these bilateral lesions, allowing better functioning of certain verbal functions. It has been reported that bilateral language networks underlie the function of some high-level language-related cognitive functions [32, 33] and single-word comprehension [34]. The splenium of the corpus callosum contains the fibres connecting the temporal lobes’ language regions [35, 36]. Consistently, adolescents with language impairments who were born preterm present with reduced splenial fibres connecting the temporal lobes [37].

White matter (WM) injury is a very common lesion pattern in spastic motor type CP [38], due to the destruction of WM fibres. A review by Scheck et al. in 2012 pointed that the evidence for the involvement of the corpus callosum in CP was conflicting; while some studies showed FA reductions in patients with CP, others did not. Since then, additional studies have showed evidence of reduced WM integrity in children with unilateral CP [25, 39, 40] and the corpus callosum has also been found to play a role in upper-extremity function in children with unilateral spastic CP [41–44].

Adding to this, diffuse thinning of the corpus callosum in nonverbal children with severe CP appears to be associated with poor spoken language comprehension [45]. A second commissural pathway that connects the temporal lobes is the anterior commissure which predominantly connects the anterior regions [46, 47]. In adolescents born preterm, language impairment is present only when both the anterior commissure and the corpus callosum temporal lobe fibre connections were reduced in size [37].

To our knowledge, the association between the WM integrity of interhemispheric fibres connecting the temporal lobes and receptive vocabulary has not been studied in children with CP. In the present study we expand on our previous study by investigating microstructural properties, rather than the MRI observable lesion load. We aim to explore the association between receptive vocabulary and 1) FA/MD in the right and left temporal lobes; 2) the FA/MD laterality index for the temporal lobes and 3) the structural connectivity between temporal lobes.

## 2. Materials and methods

### 2.1. Participants

Participants were recruited as part of a cohort study of participants with CP at the Queensland Cerebral Palsy and Rehabilitation Research Centre at the University of Queensland (Brisbane, Australia), the “Comprehensive surveillance to PREDICT outcomes for school age children with CP” (PREDICT-CP; recruitment period 2016–2020) [48]. The University of Queensland and Children’s Health Queensland ethics committees granted ethical approval (HREC/14/QRCH/329; HREC/11/QRCH/35). Informed written parental consent was obtained for all participants.

**Fig 1:**
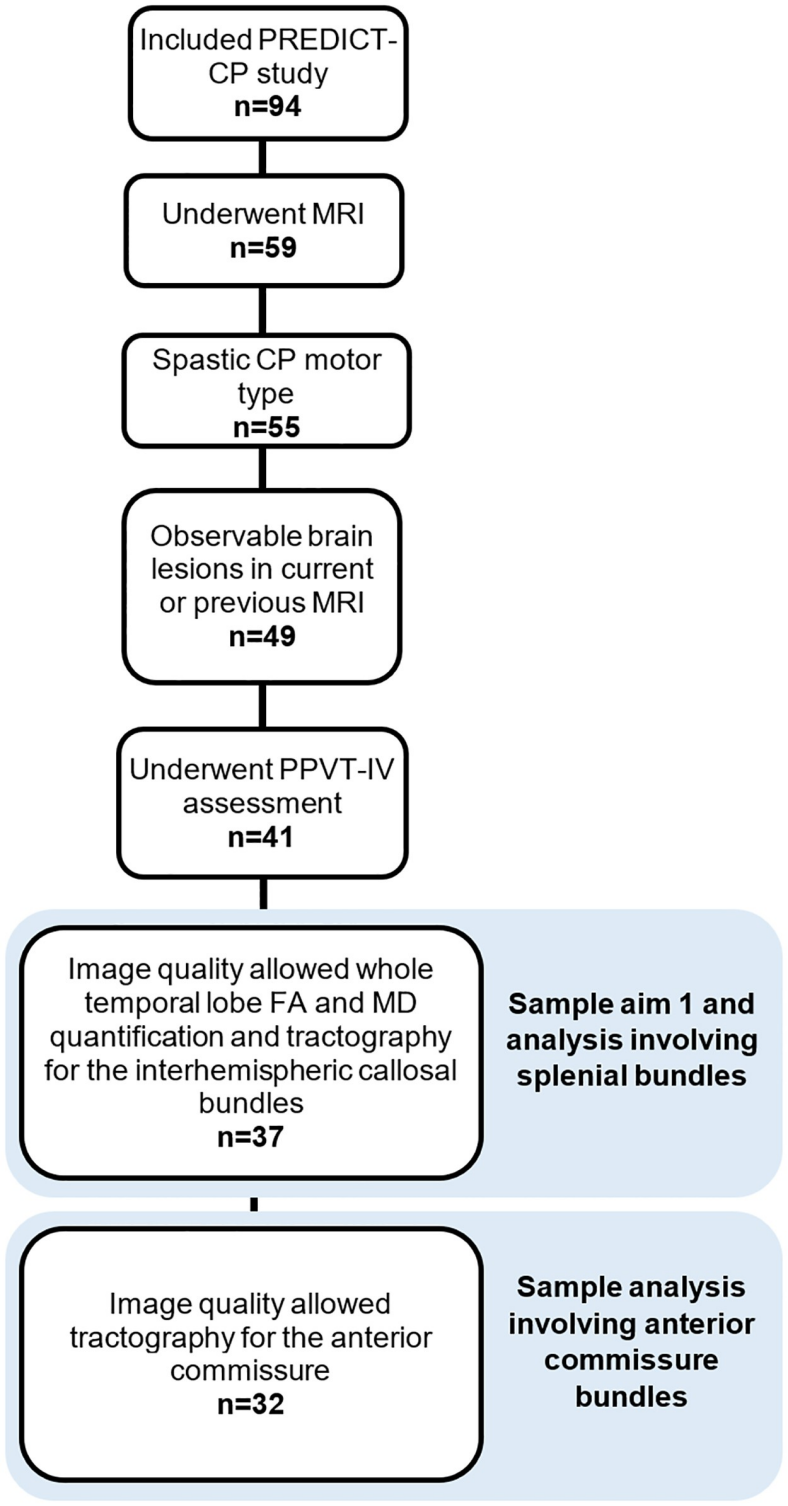
Flowchart presenting the selection of participants for each aim of the study. FA: fractional anisotropy; MD: mean diffusivity; PPVT-IV: Peabody Picture Vocabulary Test 4^th^ edition.

The present study includes 37 participants recruited as part of this study who presented with spastic CP motor type, underwent the receptive vocabulary assessment, and presented with observable brain lesions in the current or previous clinical brain MRI. Fig 1 shows the selection process for participants included in the different aims of the present study.

### 2.2. Receptive vocabulary

Receptive (hearing) vocabulary was assessed by the 4^th^ version of Peabody Picture Vocabulary Test (PPVT-IV), a proxy for verbal IQ [49]. This commonly used standardized test comprises 228 items equally distributed across 19 item-sets. Each item-set contains 12 items of increasing difficulty with 4 illustrations, and the examiner presents a word verbally to the examinee. The examinee must select the image that best represents the meaning of the word provided orally. For each correct answer one point is scored and raw scores are converted into IQ scores using normative data provided by the test. Normal standard deviation IQ-scores (mean 100; standard deviation 15) controlling for age were used. The PPVT-IV provides a proxy for verbal IQ and is considered as a screening test of intellectual functioning [49]. As it does not rely on verbal responses it is appropriate for those cases with severe expressive language impairment [50] and it has been widely used in CP [51].

### 2.3. Neuroimaging

#### 2.3.1. Image acquisition

MRI data were acquired at study enrolment using a 3T Siemens Magnetom PRISMA. Imaging included 3D T1-weighted Magnetisation Prepared Rapid Gradient Echo (MPRAGE) and diffusion weighted imaging. The MPRAGE scanning parameters were TR = 1900 ms, TE = 2.98 ms, TI = 900 ms, flip angle = 9 degrees, resolution = 1.0x1.0x1.0 mm.

Multi-shell diffusion MRI data were acquired at 2 b-values: 20 directions at b = 1000 s/mm^2^ and 64 directions at b = 3000 s/mm^2^, along with 12 non-diffusion-weighted images. Diffusion encoding directions were uniformly distributed within each shell. Directions and b-values were acquired in an order that resulted in near-optimal distribution in case of truncation. The full diffusion protocol was divided into 4 unique blocks of equal length, with 2 blocks acquired with phase encoding along anterior to posterior and 2 blocks acquired with phase encoding along posterior to anterior. This implementation enabled (i) repetition of individual blocks if excessive motion was observed, and (ii) correction for susceptibility-induced image distortions using reverse phase-encoded images. Other acquisition parameters were: TR/TE 4700/84ms, multi-band factor 2, resolution 2 x 2 mm, slice thickness 2 mm. Acquisition time of each block was 2:07 minutes, i.e. 8:28 minutes total.

#### 2.3.2. Semi quantitative lesion scoring

Brain lesion severity was assessed using the semi-quantitative scale for structural MRI (sqMRI) to better understand participants’ observable brain lesion characteristics. The sqMRI scale is valid [52] and reliable [53] and has been utilised for studies examining brain lesion severity and its association with clinical outcomes in CP [30, 54–58]. Descriptive data as scored by an MRI-trained neurologist (SF) was reported. In brief, this system scores lesion severity by brain region, where progressively higher scores represent increased lesion extent. The global sqMRI score is the total sum of all scores, ranging from 0 to 48. Each lobe is divided into three layers, periventricular, middle and cortico/subcortical and each layer is scored separately by comparing the number of slices in which the lesion encroaches on it (affected layers) to the number of slices in which the layer was represented (total layers). The lobar scores are calculated as the sum of the scores of the three layers, ranging from 0 to 3 for each hemisphere. Among other structures, the corpus callosum is assessed in three portions: the anterior, middle, and posterior corpus callosum, each scored as either a 0 (no lesion) or 1 (affected). In addition, brain lesion types were classified according to Krägeloh-Mann [59] as per previous qualitative neuroimaging studies [60–62].

#### 2.3.3. Structural image processing and segmentation

Intensity bias in the structural MRI was corrected using the N4 algorithm [63], while image de-noising, using anisotropic diffusion [64], was performed with the Insight Toolkit (ITK). Skull stripping was performed using an in-house algorithm [65] developed in MATLAB (Mathworks, Natick, MA). In this approach, intradural cerebrospinal fluid was identified using thresholding and morphological operations. This approach is capable of accurately segmenting the brain in cases of large lesions, which may be present in children with CP.

Tissue segmentation was performed using the Expectation Maximization (EM)/Markov Random Fields (MRF) [66] on the T1w MRI, which unlike atlas-based approaches are more robust to severe pathology that did not match a normative atlas. Regions of the cortex were parcellated according to the Automated Anatomical Labelling atlas labels, using the level set method [65] to match corresponding cortical regions between the participant and atlas.

#### 2.3.4. Diffusion image processing

Diffusion MRI data were cropped tightly around the head to reduce processing times. Images were denoised using Marchenko-Pastur PCA [67] implemented in MRtrix3 [68]. Individual volumes that were corrupted by head motion such that the volume was no longer self-consistent were automatically identified using a registration-based approach [69], and removed from further processing. Image distortions due to susceptibility inhomogeneities were reduced by calculating a field map from a pair of reverse phase-encoded non-diffusion-weighted images using FSL TOPUP [70], which was then applied to correct all volumes. Head motion between volumes was corrected using rigid registration implemented in MIRORR [71]. Finally, slices affected by signal dropout due to head motion were identified [72] and replaced prior to applying the rigid transformation. Slices affected by signal dropout were replaced using predicted signal intensities obtained using a spherical harmonic fit of the remaining unaffected data of the same shell (b-value) in this slice location.

Diffusion tensors were estimated separately for each shell using iterated weighted least-squares of the log-signal [73], as implemented in MRtrix3. Fibre orientation distributions were estimated using multi-shell multi-tissue constrained spherical deconvolution [74], as implemented in MRtrix3.

#### 2.3.5. Integrity of white matter in the temporal lobe

**Fig 2:**
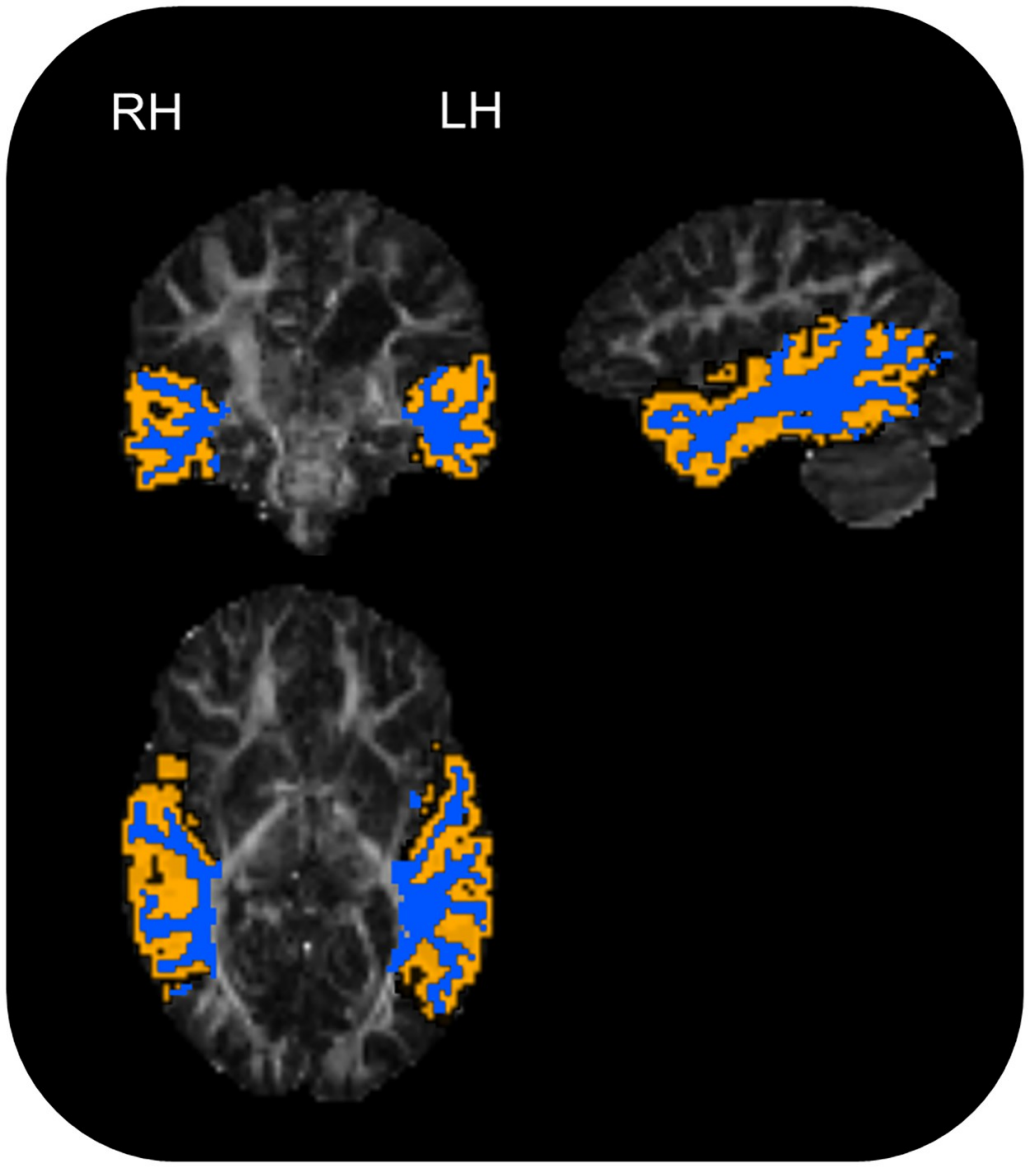
Example of white matter (in blue) and white matter/grey matter interface (in orange) labelling of the temporal lobe in a participant with periventricular white matter lesion. Sagittal slice shows left hemisphere. LH: Left hemisphere; RH: Right hemisphere.

Diffusion and structural images were rigidly aligned using boundary-based registration [75], as implemented in FSL. The tissue segmentations and parcellations were transformed to diffusion space with nearest neighbour interpolation. The tissue segmentation was then automatically ‘cleaned’ by removing WM labels in voxels with FA < 0.1 and removing grey matter voxels within one voxel (26-connectivity) of the ventricles label. To label white-matter voxels with their corresponding cortical region, the cortical grey matter parcellation was dilated ten voxels using fslmaths [76] and cropped to voxels labelled as WM by the cleaned tissue segmentation. The temporal lobe white-matter labels were then visually checked for anatomical correctness. An example of this WM labelling can be found in Fig 2. Mean FA and MD were calculated from temporal lobe WM for each hemisphere separately using mrstats [68].

#### 2.3.6. Tractography

Interhemispheric bundles traversing (a) the splenium of the corpus callosum or (b) the anterior commissure and connecting the temporal lobes were generated using MRtrix’ iFOD2 probabilistic tractography algorithm.

**Fig 3:**
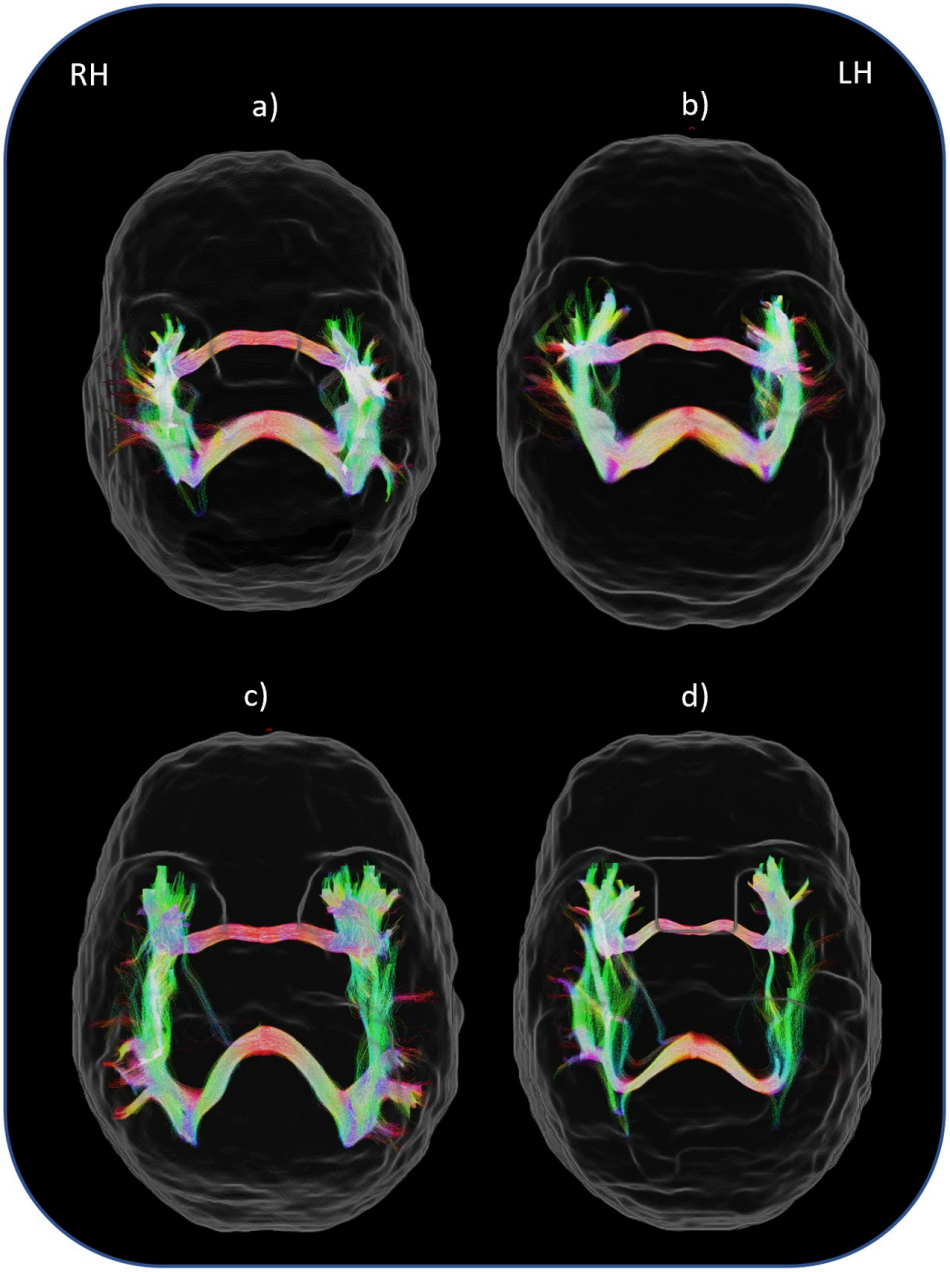
Examples of the generated tractography for four participants. LH: Left hemisphere; RH: Right hemisphere. Characteristics of the participants were: a) 8 years 1 month, GMFCS I, bilateral tetraplegia; b) 8 years 10month, GMFCS I, unilateral (right side); c) 11 years 2 months, GMFCS II, bilateral diplegia; d) 8 years 10 months, GMFCS III, unilateral (left side).

Streamlines were seeded from a region of interest (ROI) (the anterior commissure or splenium of the corpus callosum) in the mid-sagittal slice, or the slice closest to the mid-sagittal slice if the anatomy was not clear on the mid-sagittal slice. These ROIs were manually traced on the FA images, with the splenium of the corpus callosum ROI being guided by the previously detailed parcellation. The inclusion (and termination) ROI was the GM/WM interface of the temporal lobe, calculated directly from the parcellation and segmentation. We opted to perform tractography of the left and right hemispheres separately as this proved substantially more computationally efficient than tracking bilaterally, with no visible impact on the quality of the resulting tractography. When delineating the left hemisphere, the entire right hemisphere was an explicit exclusion mask, and vice versa. A manually drawn exclusion ROI was used for both corpus callosum and anterior commissure tracts to prevent streamlines passing through the occipital, frontal and parietal lobes. Anterior-commissure streamlines also explicitly excluded the fornix, as delineated by the automatic parcellation. All streamlines had a maximum length of 125mm on either hemisphere. All other settings were left at default values. For each hemisphere a minimum of 5000 streamlines were generated and the resulting tractograms were combined using MRtrix’ tckedit [20]. Finally, an automated tractography cleaning procedure removed all streamlines that traversed voxels visited by only a single streamline. An example of the tractography generated can be found in Fig 3.

Mean FA and MD of the interhemispheric connections were measured at the level of the splenium of the corpus callosum or the anterior commissure to avoid the confounding effects of crossing fibres that are in voxels further from the mid-sagittal plane [20]. Specifically, tractography was cropped to within 6 voxels (12mm) on either side of the mid-sagittal plane before metrics were taken. Mean FA and MD were also measured for the full tract, with results presented only in Supporting information because these measurements are affected by crossing fibres.

#### 2.3.7. Statistics

The associations between PPVT-IV scores and mean FA or MD in the temporal lobe were explored with univariable linear regressions (Aim 1). FA/MD temporal lobe laterality indices were similarly regressed against PPVT-IV scores univariably (Aim 2). FA/MD temporal lobe laterality indices ranged from -1 to 1 with a score approximating zero indicating a more similar WM microstructure between temporal lobes; a score approaching ‘one’ indicating higher right-sided FA/MD values and a score approaching ‘negative one’ indicating lower right-sided FA/MD values. Finally, the associations between mean FA/MD around the midline in the commissural bundles connecting the temporal lobes and PPVT-IV scores were explored with univariable linear regression models (Aim 3).

Age and sex were not significantly associated with any brain measure used in the analysis. Given that, and that the PPVT-IV scores are age-adjusted, age and sex were not included as a covariable in our analysis. The normality of the residuals was checked by using scatterplots, histograms and the Kolmogorov-Smirnov test. No datapoints were rejected as outliers. Significance level was set at 0.05 and data were analysed with the Statistical Package for the Social Sciences (SPSS, v27.0) [77]. Multiple comparisons correction was not performed because FA and MD index assess different aspects of WM microstructure–MD the absolute average diffusivity and FA a unitless ratio of diffusivity of the major fibre orientation relative to others–and so are interpreted here differently.

## 3. Results

### 3.1. Group characteristics

**Fig 4:**
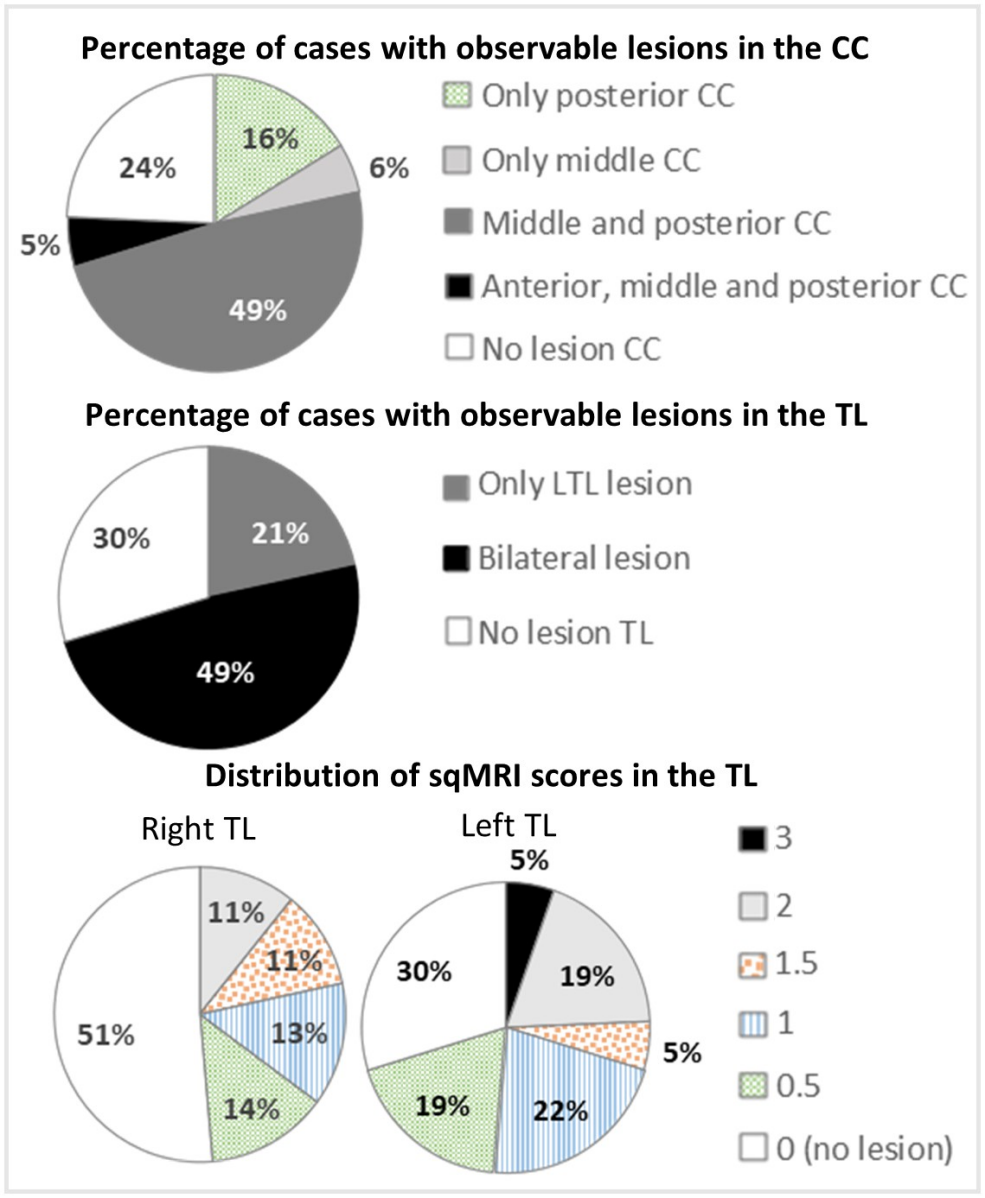
Percentage of cases presenting an observable lesion in different sections of the corpus callosum (top row) and right, left, or both temporal lobes (middle row). The distribution of semi-quantitative scale scores in the right and left temporal lobe are also presented (bottom row). Higher scores represent increased lesion extension. CC: corpus callosum; LTL: left temporal lobe; TL: temporal lobe.

Demographic and clinical characteristics of the sample are presented in Table 1. Most participants did not present with epilepsy (95%) and were at the level I of the GMFCS (70%) and the MACS (51%). Most participants were preterm (65%) and presented with unilateral CP (54%) but bilaterally distributed lesions were more frequent (68%). It is important to note that our sample included only two participants with unilateral right-sided brain lesions, and nine with unilateral left-sided brain lesions. Fig 4 presents the sqMRI scores’ descriptive statistics for the temporal lobe and corpus callosum. This information is not provided for the anterior commissure because the sqMRI scale does not provide an independent score for the anterior commissure. Most participants (74%) presented with a lesion in the corpus callosum, with 70% presenting lesions involving the posterior region. When lesions were present in the temporal lobe (70%), they were bilateral (49%) or circumscribed to the left side (21%). The movement identified during the pre-processing was relatively low, with a mean of only two volumes of 92 required scrubbing of motion corrupted volumes (range 0–16, median = 0, interquartile range = 3).

**Table 1:** Demographics and clinical characteristics of the study sample (n = 37).

**General demographics**	
Sex n female / male	12 / 25
Age Mean (SD) / range	9.59 (0.19) / 9–12
**Gestational age**	
Term / preterm	13 / 24
**Motor functioning**	
n unilateral (left / right side affected hemiparesis) / bilateral (diplegia / triplegia / quadriplegia)	(7/13) / (13/1/3)
GMFCS, levels (n)	I (26); II (7); III (3); IV (1)
MACS, levels (n)	I (19); II (14); III (4)
**Brain lesion**	
Bilateral / unilateral right-sided / unilateral left-sided / no observable lesion	25 / 2 / 9 / 1
n PWM / CDGM / brain maldevelopment / normal MRI	31 / 3 / 2 / 1
sqMRI global score Mean (SD) / range	9.9 (5.5) / 0–20
**White matter microstructure**	
Mean (SD) FA / MD right temporal lobe	0.36 (0.03) / 631x10^-6^ (18.3x10^-6^)
Mean (SD) FA / MD left temporal lobe	0.36 (0.03) / 631x10^-6^ (24.5x10^-6^)
Mean (SD) FA / MD splenium bundle	0.66 (0.08) / 656x10^-6^ (50x10^-6^)
Mean (SD) FA / MD anterior commissure bundle	0.34 (0.07) / 638x10^-6^ (36.2x10^-6^)
**Receptive vocabulary**	
PPVT-IV score	99.32 (16.06)
**Handedness Edinburgh Handedness Inventory (n)** [78]	right-handed (16); left-handed (18); ambidextrous (3)
**Epilepsy status**	
n no epilepsy / epilepsy	35/2

Key: CDGM: cortical and deep grey matter; FA: fractional anisotropy; GMFCS: Gross motor function classification system; MACS: Manual ability classification system; MD: mean diffusivity; PPVT-IV: Peabody Picture Vocabulary Test; PWM: Periventricular white matter; SD: standard deviation

### 3.2. Association between receptive vocabulary and mean whole temporal lobe WM FA/MD as well as FA/MD laterality index

Results from the linear regression models relating to Aims 1 and 2 are presented in Table 2. PPVT-IV scores were not significantly associated with mean WM FA in the temporal lobe in the right (β = -0.15, p = 0.37) or left hemisphere (β = 0.09, p = 0.59). PPVT-IV scores were significantly associated with mean WM MD in the left temporal lobe (β = -0.33, p = 0.04) but not in the right temporal lobe (β = -0.28, p = 0.10). Finally, PPVT-IV scores were not significantly associated with the FA/MD laterality indices (β = -0.26, p = 0.12 and β = -0.17, p = 0.31 respectively).

**Table 2:** Univariable association between receptive vocabulary and FA/MD measures in the WM of the whole temporal lobe.

Dependent variable	Independent variables		Right temporal lobe	Left temporal lobe
PPVT-IV scores	FA	R^2^	0.02	0.01
		p	0.37	0.59
		B (95% CI)	-96.64 (-311.19, 117.92)	43.99 (-120.38, 208.35)
	MD	R^2^	0.08	0.11
		p	0.10	0.04*
		B (95% CI)	-245x10^3^ (-534x10^3^, 44.7x10^3^)	-218x10^3^ (-430x10^3^, -6.36x10^3^)
			Bilateral	
	FA laterality index	R^2^	0.07	
		p	0.12	
		B (95% CI)	-101.50 (-230.36, 27.35)	
	MD laterality index	R^2^	0.03	
		p	0.31	
		B (95% CI)	-201.76 (-194.41, 597.93)	

Key: B: regression coefficient;

*p ≤ 0.05;

FA: fractional anisotropy; MD: mean diffusivity; CI: confidence interval

### 3.3. Association between integrity of interhemispheric fibres and PPVT-IV scores

FA, but not MD, in the central part of the temporo-temporal bundles traversing the splenium of the corpus callosum was significantly associated with the PPVT-IV scores (β = 0.34, p = 0.04 and β = -0.22, p = 0.18, respectively). Neither FA nor MD in the central part of the temporo-temporal bundles traversing the anterior commissure where significantly associated with the PPVT-IV scores (β = -0.07, p = 0.69 and β = -0.02, p = 0.91 respectively) (Table 3). The association between FA and MD for the full tracts and PPVT-IV scores is presented in S1 Table. No association reached significance when considering the whole bundles. Consistently with our results for the central part of the bundles, the association between FA in the splenium of the corpus callosum and the PPVT-IV scores was the association closer to significance (p = 0.11).

**Table 3:** Univariable association between receptive vocabulary and FA/MD measures in the central part of the temporo-temporal bundles.

Dependent variable		Independent variable		
PPVT-IV scores	Corpus callosum	FA	R^2^	0.12
			p	0.04*
			B (95% CI)	66.47 (3.31, 129.64)
		MD	R^2^	0.05
			p	0.18
			B (95% CI)	-72.2x10^3^ (-180x10^3^, 35.8x10^3^)
	Anterior commissure	FA	R^2^	0.01
			p	0.70
			B (95% CI)	-15.22 (-94.69, 64.25)
		MD	R^2^	<0.01
			p	0.91
			B (95% CI)	-8.54x10^3^ (-167x10^3^, 150x10^3^)

Key: B: regression coefficient;

*p ≤ 0.05;

FA: fractional anisotropy; MD: mean diffusivity; CI: confidence interval

## 4. Discussion

In this study we explored in depth the association between receptive vocabulary skills and WM microstructure within the temporal lobes and the commissural fibres connecting them in children with spastic motor type CP. We found that the lower the MD of the left temporal lobe, the better the receptive vocabulary performance. Vocabulary performance was not significantly associated with mean FA in the temporal lobe WM nor mean MD in the right temporal lobe WM. PPVT-IV scores were also not significantly associated with the laterality of these FA/MD indices. Within the corpus callosum, FA, but not MD, of the temporo-temporal bundles were positively and significantly associated with the PPVT-IV scores. Within the anterior commissure no equivalent relationship between diffusion metrics and PPVT-IV was found.

Despite clear patterns of injury, our participants presented with a mean standardized PPVT-IV score within the normal limits (see Table 1). This is consistent with the brain’s ability to reorganize language areas [26] and to retain or regain performance to within the normal limits after early purely unilateral brain lesions [79]. That said, the brain’s reorganising potential is not limitless. Our results highlight that the left temporal lobe status, as previously reported [79, 80], but not the right temporal lobe status can still affect performance to some degree. The association found between MD of the left temporal lobe and receptive vocabulary ratifies the importance of left temporal lobe microstructure for verbal functions in children with CP [30]. It is also consistent with our previous study in a larger sample (which includes all subjects from the present study) where we found that temporal lobe lesions being distributed toward the left, rather than the right, hemisphere was associated with poorer receptive vocabulary [31].

Unlike the significant association between the lesion temporal lobe laterality index and PPVT-IV found in our previous study [31], here the laterality indices for FA and MD were not significantly associated with the performance in the PPVT-IV. This is most likely due to two reasons. Firstly, the lesion load, as measured by the sqMRI in our previous study, is a measure that accounts for the most visually damaged tissue, while tractography and WM FA/MD measurement predominantly considers visually intact tissue. Visually intact tissue does not mean completely intact tissue. Rather, tissue that appears intact on a standard structural scan has not been so heavily damaged that it drastically affects T1 and T2 signals. Secondly, FA/MD of the temporal lobe WM cannot measure hemispheres as independently as the sqMRI scores. This is because these metrics combine signal from intra- and inter-hemispheric fibres. Acute infarcts in one hemisphere can be expected to cause primary (destructive) or secondary (degeneration, but also abnormal pruning and connectivity after the brain lesion) abnormalities in connectivity [81], potentially affecting FA/MD in the other hemisphere.

The corpus callosum, and especially its posterior part, is a common region involved in our sample. This has also been identified in several diffusion studies but not all [21, 25, 39, 40]. In the present study, we were unable to compare tensor metrics of our sample with a control group. We found, however, that within the splenium of the corpus callosum, FA, but not MD, in the temporo-temporal bundle was significantly associated with the PPVT-IV scores. Although the association was only of a medium effect size, this is a key finding consistent with Geytenbeek et al. [45] findings indicating that the thinning of the corpus callosum was associated poor spoken language comprehension in CP. Our results also align with theories which suggest that interhemispheric connections are important for specific elements of language, including word comprehension [34]. For example, Catani et al. [32] found that the degree of lateralization of perisylvian pathways is heterogeneous in the normal population and that bilateral representation might be advantageous for remembering words using semantic association. Our results align with those in populations of children born preterm (note that in our sample 64.9% of participants were born preterm). That is, interhemispheric temporal lobe connectivity is a robust predictor of language impairments in preterm born adolescents not presenting with neurological diagnoses [37] and the area of splenium in very preterm born adolescents (some of whom presented with a neurological diagnosis) is associated with vocabulary performance [82].

Consistently, in healthy children, larger volumes of the posterior parts of the posterior corpus callosum have been shown to be associated with better vocabulary and increased interhemispheric language network connectivity [83]. This seems to be linked with the finding that bilateral mesial temporal lobe involvement is advantageous for vocabulary skills in healthy, right-handed children [84].

We found a significant association with MD, but not FA, for the whole left temporal lobe while finding the reverse for the temporo-temporal bundles within the splenium of the corpus callosum. It is likely that this reflects the strengths and limitations of these measures. Specifically, the calculation of FA from the diffusion tensor model is driven by the majority direction of fibres in each voxel, while the calculation of MD is simply the average diffusivity. Within the corpus callosum where WM fibres are unidirectional (i.e. high FA), FA may provide a more sensitive measure of microstructure than MD. By contrast, in the temporal lobe voxels with predominantly contain crossing fibres [20], MD is the more optimal and interpretable measure as it does not rely on correct identification of the major fibre orientation, nor that the major fibre pathway is dedicated to language function.

Within the anterior commissure, no equivalent relationship between diffusion metrics and PPVT-IV was found. The association between the size of the anterior commissure and severity of language impairments has been described in preterm adolescents who also presented with volume reductions in the splenium of the corpus callosum [37]. Interestingly, in this study language impairment was only present if the volumes of the anterior commissure and the splenium of the corpus callosum were both reduced. This has been discussed as an indication that the anterior commissure may provide some compensation for splenium damage. This is also supported by cases of callosal agenesis or callosotomy where it has been suggested that the anterior commissure might partially take over the interhemispheric connection function, compensating for the lack of interhemispheric communication via the corpus callosum [85]. It is possible that our sample’s WM integrity in the splenium was sufficient to not require compensatory reliance on the anterior commissure. This is however a hypothesis that is difficult to assess in the current study because our study lacked healthy control participants with MRI studies on the same scanner, preventing us from knowing the absolute degree to which the WM microstructure in the splenium of the corpus callosum was affected. The sqMRI score, however, provides an approximate measure of the status of the splenium of the corpus callosum and indicates that lesions were observable in the splenium in 70% of the cases. It is also notable that measuring the anterior commissure is very challenging: its small size combined with limited spatial resolution of diffusion images can mean measurements are considerably affected by partial volume effects, reducing signal to noise and thus statistical power.

Although the main limitation of the present study is its moderate sample size, it is sufficient to identify effects as small as 0.18 in the generated linear regression models with 80% confidence. Our current sample has value amongst existing literature as samples of this size and type are unusual in the CP literature because neuroimaging and neuropsychological assessments are extremely challenging to conduct in this population. Another limitation is that language lateralization was not confirmed in our participants and having such information would allow a more comprehensive interpretation of our results. Additionally, it must be remembered that CP is a heterogenous group of conditions, and different mechanisms of recovery are known for several functions between participants with bilateral or solely unilateral brain lesions. Given our cohort (Table 1), results presented here are likely to generalise best to patients who have bilateral periventricular WM lesions. Future studies with larger samples that allow more complex analyses should ideally consider grouping participants by relevant variables such as being term/preterm born or the lesion type, which are likely to play a role in the relationship between brain structure and clinical outcomes.

## 5. Conclusion

The findings of our study add further understanding to the pathophysiological bases underlying receptive vocabulary skills in children with CP that could extend to other patients with early brain damage. This study complements our previous findings that used a semiquantitative measure of brain lesion severity and highlights the importance of interhemispheric connections for receptive vocabulary. This knowledge contributes to the ultimate goal of better predicting early in life cognitive functioning based on brain characteristics in order to provide early and individualized interventions.

## Supporting information

S1 Table Univariable association between receptive vocabulary and FA/MD measures in the whole temporo-temporal bundles. B: regression coefficient; *p ≤ 0.05; FA: fractional anisotropy; MD: mean diffusivity; CI: confidence interval.
(PDF)

S1 Data Spreadsheet including all relevant numerical data. (XLSX)

## Abbreviations

CP: Cerebral palsy
FA: fractional anisotropy
MD: mean diffusivity
MRI: magnetic resonance imaging
PPVT-IV: Peabody Picture Vocabulary Test
ROI: region of interest
WM: white matter
