# Supplementary material for: White matter microstructure and receptive vocabulary in children with cerebral palsy: The role of interhemispheric connectivity
Source: PLoS One. 2023 Jan 17;18(1):e0280055. doi: 10.1371/journal.pone.0280055 (PMC9844879; doi:10.1371/journal.pone.0280055)
Supplement: S1 Table — B: regression coefficient; *p ≤ 0.05; FA: fractional anisotropy; MD: mean diffusivity; CI: confidence interval. (PDF) [file pone.0280055.s001.pdf]

S1 Table. Univariable association between receptive vocabulary and FA/MD measures in the whole temporo-temporal bundles

Dependent variable		Independent variable		
PPVT-III scores	Corpus callosum	FA	R^2	0.07
			p	0.11
			B (95% CI)	103.89 (-25.47, 233.24)
		MD	R^2	0.02
			p	0.45
			B (95% CI)	-4.62×10^4 (-1.71×10^5, 7.87×10^4)
	Anterior commissure	FA	R^2	1.22×10^4
			p	0.95
			B (95% CI)	-3.6 (-125.38, 118.17)
		MD	R^2	0.02
			p	0.47
			B (95% CI)	-9.18×10^4 (-3.49×10^5, 1.66×10^5)
